# Low satisfaction with normative life domains in adolescents with anorexia nervosa

**DOI:** 10.1002/cpp.2574

**Published:** 2021-03-02

**Authors:** Sanne F. W. van Doornik, Brian D. Ostafin, Nienke C. Jonker, Klaske A. Glashouwer, Peter J. de Jong

**Affiliations:** ^1^ Department of Clinical Psychology and Experimental Psychopathology University of Groningen Groningen Netherlands; ^2^ Department of Eating Disorders Accare Child and Adolescent Psychiatry Groningen Netherlands

**Keywords:** adolescence, anorexia nervosa, goals, life domains, meaning in life, satisfaction

## Abstract

**Objective:**

Low satisfaction with normative life domains might be an important factor in the persistence of anorexia nervosa (AN). Initial evidence in non‐clinical samples showed that lower satisfaction with normative life domains was related to more intense eating disorder symptoms. As a critical next step, the current study examined satisfaction with normative life domains in a clinical sample. Specifically, the present study tested whether adolescents with AN reported lower satisfaction with normative life domains than adolescents without an eating disorder.

**Method:**

Adolescents with AN (*n* = 69) and adolescents without an eating disorder matched on age, gender and educational level (*n* = 69) completed the Brief Multidimensional Students' Life Satisfaction Scale to assess satisfaction with five life domains (family, friendships, school, self and living location) and life in general.

**Results:**

Adolescents with AN reported significantly lower satisfaction with normative life domains than the comparison group. Subsequent analyses showed that this overall group difference was primarily driven by adolescents with AN reporting lower satisfaction with the self, school experience and life in general.

**Conclusions:**

Findings supported the hypothesis that adolescents with AN show relatively low satisfaction with meaningful, non‐AN‐related life domains. This points to the potential relevance of enhancing satisfaction with specific life domains to optimize treatment effectiveness.

**Key Practitioner Message**

- Adolescents with AN were less satisfied with normative life domains, compared to adolescents without an eating disorder.
- In particular, adolescents with AN were less satisfied with themselves, with their school experiences and with life in general.
- Addressing low satisfaction with these life domains may be beneficial in optimizing treatment effectiveness.

## 1 INTRODUCTION

Anorexia nervosa (AN) is a severe mental disorder, characterized by an extreme restriction of food intake leading to significant weight loss and an intense fear of gaining weight (American Psychiatric Association, 2013). AN has severe physical consequences and a high burden of disease for individuals with AN, their relatives and society (Schmidt et al., 2016). Despite the great impact of the disorder, treatment options are limited in their effectiveness, as many individuals with AN do not respond to treatment, drop‐out during treatment or relapse after successful treatment (e.g., Berends et al., 2018; Murray et al., 2019). Therefore, it is important to broaden our knowledge of the factors that are important in the maintenance of AN. In the present study, we focussed on satisfaction with normative life domains.

According to an influential cognitive behavioural theory of eating disorders, most of the clinical features seen in AN can be understood as stemming directly from the excessive influence of body weight and shape on self‐evaluation (Fairburn et al., 2003). In contrast to most people whose lives are guided by values and goals in different life domains (e.g., work, school and relationships), individuals with AN seem to primarily concern themselves in regards to values related to weight, shape and eating behaviour (Fairburn et al., 2003). This overvaluation may create a self‐reinforcing loop, in which the goal to lose weight enhances the motivational salience of goal‐related cues (e.g., ‘fat’ body parts), which causes these cues to grab attention and to be processed more elaborately, which in turn reactivates the goal to lose weight again (Fairburn et al., 2003; Williamson et al., 2004). It has been hypothesized that, as a consequence, the salience landscape (i.e., to what extent environmental stimuli elicit appetitive or aversive motivation) and goal‐directed activity of individuals with AN may increasingly narrow to weight and shape‐related concerns.

The pursuit of important goals, together with the feeling that life makes sense and that one matters in the world, is thought to play a central role in whether individuals experience their lives as meaningful (George & Park, 2016; Martela & Steger, 2016). In addition, theory and empirical findings suggest that the experience of life meaning depends on the ability to engage with one's goals in a positive, satisfying way (King et al., 2006; Wolf, 2010). According to Fox and Leung (2009), AN may serve as a maladaptive attempt to deal with a sense of purposelessness or existential anxiety. That is, in the short run, AN may provide individuals with goals and feelings of purpose (Ison & Kent, 2010; Serpell et al., 1999) and thus a way of imposing meaning upon their world (Fox & Leung, 2009). However, the narrowed salience landscape and goal‐directed activity of individuals with AN (i.e., striving to achieve AN‐related goals) may lead to a tendency to neglect or compromise more overarching personal values and common sources of life meaning (Mulkerrin et al., 2016), including interpersonal relationships, personal development and generativity (Emmons, 2003; Schnell, 2011). Therefore, in the long run, AN may create a myopic perspective that prevents access to a wide range of normative goals and domains that provide meaning across time and situations (Marco et al., 2017).

Although excessive engagement with weight and shape‐related goals is seen as a core mechanism of AN (Fairburn et al., 2003), relatively little research has examined the role that a lack of engagement and satisfaction with normative, non‐AN‐related goals might play in the disorder. The only two clinical studies on meaning in life and eating disorder pathology (Marco et al., 2017; Marco et al., 2019) have used the Purpose in Life Questionnaire (Crumbaugh & Maholick, 1969), which cannot differentiate satisfying engagement with normative goals from satisfying engagement with AN‐related goals (with items as ‘In life I have no goals nor aims at all’). This could be problematic, as measures that do not differentiate between these types of goals could potentially show high purpose in individuals with AN, due to satisfactory engagement in dysfunctional AN‐related goals (Ison & Kent, 2010; Serpell et al., 1999).

Several studies can be found demonstrating that individuals with AN report lower levels of satisfaction with life as a whole than individuals without an eating disorder (Garcia et al., 2017; Kitsantas et al., 2003; Magallares et al., 2014). However, thus far, only one study has examined the relationship between eating disorder symptoms and satisfaction with normative, non‐AN‐related domains (Matthews et al., 2012). In this study, a non‐clinical student sample completed the college version of the Brief Multidimensional Students' Life Satisfaction Scale (BMSLSS; Seligson et al., 2003), which allows the assessment of satisfaction with respect to different life domains (e.g., family life, friendships and school experience) and life in general, which are common goals or sources from which people derive meaning (Delle Fave et al., 2011). Results indicated that multiple eating disorder behaviours and weight perceptions (e.g., binge eating, worrying about weight and self‐described weight) were inversely related to satisfaction with each individual domain (Matthews et al., 2012). The strongest correlations were found between students' weight perceptions and satisfaction with the self and physical appearance, followed by satisfaction with life in general.

The study of Matthews et al. (2012) provides important initial evidence that, in a non‐clinical sample, less satisfaction with normative life domains is associated with more eating disorder symptoms. A crucial next step is to examine whether this inverse relation is also evident in individuals who are clinically diagnosed with AN. It is important to provide empirical evidence on this matter, as the overall impairment in functioning seen in AN (American Psychiatric Association, 2013) does not necessarily equate less satisfaction with normative life domains. Furthermore, the present study differentiates between satisfaction with various domains, which could provide concrete targets for interventions. Our main aim was to examine satisfaction with normative life domains in a clinical sample of young individuals with AN. The literature on general life satisfaction and (health related) quality of life in individuals with AN showed reduced quality of life in various life areas compared to healthy controls (Ágh et al., 2016; Engel et al., 2009; Sy et al., 2013). We therefore hypothesized that adolescents with AN would report less satisfaction with normative life domains compared to a comparison group of adolescents without an eating disorder. Furthermore, because the salience landscape and goal‐directed activity in individuals with AN seem to increasingly narrow to weight and shape‐related concerns (Fairburn et al., 2003), we also hypothesized that individuals with AN would report normative life domains to be less important, compared to the comparison group.

## 2 METHODS

### 2.1 Participants

Individuals with AN (*n* = 69, 67 female, *M*_age_ = 15.55, *SD* = 1.70) between the ages of 12 and 22 were recruited through the Department of Eating Disorders of Accare, a facility for child and adolescent psychiatry in the Netherlands, between June 2015 and June 2017. Eating disorder pathology was assessed by health care professionals of Accare using the Dutch child version of the Eating Disorder Examination (EDE) interview (Bryant‐Waugh et al., 1996; Jansen et al., 2007). Most individuals with AN presented with their first episode of an eating disorder (*n* = 62) and a minority presented with a second episode (*n* = 7). Individuals with AN met DSM‐5 criteria for AN (Restrictive subtype *n* = 39; Binge Purge subtype *n* = 10) or atypical AN (i.e., ‘all of the criteria for anorexia nervosa are met, except that despite significant weight loss, the individual's weight is within or above the normal range’; American Psychiatric Association, 2013, p. 353; Restrictive subtype *n* = 11; Binge Purge subtype *n* = 9), thus, in the current study, AN was broadly defined. No additional inclusion or exclusion criteria were applied. Participants for the comparison group were recruited at two high schools (*n* = 62) and a secondary vocational college (*n* = 7) in the northern part of the Netherlands. Short oral presentations about the current study were given to several groups of students, which were chosen based on the characteristics of the individuals with AN (i.e., age and educational level). After the presentation participants were informed about when the assessment would take place (i.e., approximately 2 weeks later) and received an information letter and informed consent forms for themselves and their parents to take home. The final comparison group consisted of 69 healthy weight individuals without an eating disorder, who were as a group selected to be as similar as possible to the individuals with AN regarding age, gender and educational level (67 female, *M*_age_ = 15.48, *SD* = 1.82). In return for their participation, all participants received monetary compensation.

### 2.2 Materials

#### 2.2.1 Body mass index

In order to make BMI comparable over the age range of individuals included in the study (Cole et al., 2000), adjusted BMI was calculated ([actual BMI/Percentile 50 of BMI for age and gender] × 100). The 50th percentile of BMI for age and gender was derived from the Netherlands Organization for Applied Scientific Research (TNO, 2010). Adjusted BMI scores between 85% and 120% were considered to be healthy weight; scores smaller than 85% were considered to be underweight (Van Winckel & Van Mil, 2001).

#### 2.2.2 Eating disorder symptoms

A Dutch translation of the most recent version of the Eating Disorder Examination Questionnaire (EDE‐Q 6.0; Fairburn & Beglin, 2008) was used to provide a global measure of the severity of eating disorder psychopathology. The EDE‐Q is the questionnaire version of the EDE interview and assesses self‐reported eating disorder symptoms over the last 28 days. Items are answered on a scale from 0 (*no days*/*not at all*) to 6 (*every day*/*markedly*). To be suitable for administration among young individuals, adaptations to the wording of some items were made, which were in line with adaptations made for previous versions of the EDE‐Q (Jansen et al., 2007). An average score of the 22 items was used as a general index of eating disorder pathology (cf. Aardoom et al., 2012), with higher scores indicating more intense eating disorder pathology. The internal consistency of the EDE‐Q was excellent for both individuals with AN and the comparison group (*α* = .93 and *α* = .95, respectively).

#### 2.2.3 Satisfaction with and importance of normative life domains

To measure satisfaction with normative life domains, the Brief Multidimensional Students' Life Satisfaction Scale–Peabody Treatment Progress Battery version (BMSLSS‐PTPB; Bickman et al., 2007) was administered, a revision of the original BMSLSS (Seligson et al., 2003). In the BMSLSS‐PTPB, the 7‐point Terrible‐Delighted scale is replaced by a 5‐point Likert‐type scale ranging from 1 (*very unsatisfied*) to 5 (*very satisfied*) (Bickman et al., 2007). For each normative life domain included in the BMSLSS‐PTPB (i.e., family, friendships, school experience, self and living location), participants indicate how satisfied they are with it at the moment. An additional item assesses satisfaction with overall life. The responses on the six items were averaged, with higher scores indicating higher satisfaction with normative life domains (cf. Athay et al., 2012). Although the BMSLSS‐PTPB has not previously been used in individuals with eating disorders, the questionnaire has demonstrated sound psychometric qualities in other samples (Bickman et al., 2010; Büssing et al., 2009; Seligson et al., 2003; Zullig et al., 2009). In the current study, the internal consistency was satisfactory for both individuals with AN and the comparison group (*α* = .72 and *α* = .87, respectively). More item descriptive statistics can be found in Table 1.

**TABLE 1:** Item analysis and inter‐item correlations of the BMSLSS‐PTPB (satisfaction items)

Group	Satisfaction item	Skewness (*SE*)	Kurtosis (*SE*)	Corrected item‐total correlation	*α* if item deleted	1	2	3	4	5	6
AN	1. Family	−1.08 (.29)	.11 (.57)	.45	.68		.49	.27	.05	.22	.40
	2. Friendships	−.92 (.29)	−.38 (.57)	.53	.66			.35	−.01	.42	.42
	3. School	−.54 (.29)	−.80 (.57)	.46	.68				.44	.08	.42
	4. Self	1.10 (.29)	.41 (.57)	.24	.73					.07	.34
	5. Living location	−.84 (.29)	.25 (.57)	.39	.70						.48
	6. In general	.25 (.29)	−.49 (.57)	.65	.62						
Comparison	1. Family	−1.34 (.29)	.91 (.57)	.64	.86		.55	.53	.48	.45	.54
	2. Friendships	−1.54 (.29)	1.85 (.57)	.64	.86			.47	.34	.59	.59
	3. School	−.56 (.29)	−.41 (.57)	.67	.85				.65	.42	.48
	4. Self	−.31 (.29)	−.54 (.57)	.68	.85					.51	.71
	5. Living location	−1.07 (.29)	.05 (.57)	.64	.86						.89
	6. In general	−1.00 (.29)	.35 (.57)	.78	.83						

Abbreviations: AN, anorexia nervosa; Comparison, adolescents without an eating disorder.

Furthermore, in a separate set of questions, participants' importance ratings for each life domain and overall life were obtained, using a scale from 1 (*very unimportant*) to 5 (*very important*; cf. Seligson et al., 2003). The items were scored in the same manner as the aforementioned items, with higher scores indicating the life domains to be more important. The internal consistency for these items was considered to be sufficient for the individuals with AN and excellent for the comparison group (*α* = .66 and *α* = .92, respectively).

### 2.3 Procedure

The current study was approved by the medical ethical committee of the University Medical Center in Groningen in the Netherlands (NL.51694042.14). All participants, and their parents when participants were younger than 18, provided written informed consent. As part of the regular diagnostic procedure at the Eating Disorder Department of Accare, health care professionals administered the EDE interview, on which DSM‐5 classifications were based. All individuals with AN gave active consent to use this information for the present study. For individuals with AN, assessment took place at the treatment centre, at the start of treatment or up to 4 weeks after the intake (median 53 days after intake). For the comparison group, the study took place at their school. During assessment, all participants completed the EDE‐Q and BMSLSS, and their height and weight were measured. As the current study was part of a larger study other measurements were collected as well, but these are not of interest for the current study (see Jonker et al., 2019, 2020).

### 2.4 Statistical analyses

Group differences on adjusted BMI were assessed with an independent sample *t* test. Because EDE‐Q scores were not normally distributed (see the supporting information), a Mann–Whitney *U* test was used to assess group differences on the EDE‐Q. To examine differences on satisfaction with and importance of normative life domains between adolescents with broadly defined AN and those without an eating disorder, a Multivariate Analysis of Variance (MANOVA) was performed with the average satisfaction score and the average importance score as dependent factor, and group (AN vs. non‐AN‐comparisons) as fixed factor. Afterwards, univariate ANOVAs were used to examine on which variable(s) differences between groups were found. Power calculations indicated that the power of the MANOVA was .74 to detect medium‐sized effects with *α* = 0.05 and the power of the between subject test was .83.

## 3 RESULTS

### 3.1 Group characteristics

Group characteristics are presented in Table 2. As expected, individuals with AN showed lower BMI (*t*(136) = −9.74, *p* < .001, *CI* [−21.87, −14.49]) and higher EDE‐Q scores (*U* = 240.50, *p* < .001), than individuals of the comparison group.

**TABLE 2:** Group characteristics

	Individuals with AN (*n* = 69)		Comparison group (*n* = 69)	
	Mean	SD	Mean	SD
Age	15.55	1.70	15.48	1.82
BMI	84.69	12.16	102.87	9.62
EDE‐Q	4.16	1.11	1.30	1.10

Abbreviations: AN, anorexia nervosa; BMI, adjusted Body Mass Index; comparison group, adolescents without an eating disorder; EDE‐Q, mean score on the Eating Disorder Examination Questionnaire.

### 3.2 Satisfaction and importance of normative life domains

When checking the assumptions for the analyses, visual inspection showed that the assumption of multivariate normality of residuals for the mean importance score was violated to a certain degree. Additionally, two multivariate outliers (both from the comparison group, regarding mean importance score) were detected when checking Mahalanobis Distance. However, MANOVAs are relatively robust to violations of multivariate normality, especially when using relatively large sample sizes as in the current study (Finch, 2005). Furthermore, because excluding the outliers did not visually improve multivariate normality and also did not significantly change the results, a MANOVA using all participants is reported.

Table 3 shows the mean satisfaction and importance scores of each individual domain, for both groups separately. A significant difference was found between individuals with AN and the comparison group on satisfaction with and importance of meaningful life domains (*Λ* = 0.86, *F*[2,135] = 11.33, *p* < .001, *η* ^*2*^_*p*_ = 0.14). Between subjects tests showed that the overall effect was mainly due to lower satisfaction scores on the life domains measure in individuals with AN (*F*[1,136] = 21.71, *p* < .001, *η* ^*2*^_*p*_ = 0.14). Regarding the importance of meaningful life domains, no significant difference was found between both groups (*F*[1,136] = 0.18, *p* = .670).

**TABLE 3:** Mean satisfaction and importance scores per group

	Satisfaction		Importance	
	Individuals with AN (*n* = 69)	Comparison group (*n* = 69)	Individuals with AN (*n* = 69)	Comparison group (*n* = 69)
	Mean (*SD*)	Mean (*SD*)	Mean (*SD*)	Mean (*SD*)
Family	4.26 (0.95)	4.13 (1.15)	4.62 (0.77)	4.38 (1.32)
Friendships	3.80 (1.35)	4.06 (1.14)	4.54 (0.90)	4.42 (1.32)
School	3.16 (1.09)^a^	3.64 (1.07)^a^	4.28 (0.75)	4.06 (1.15)
Self	1.72 (0.89)^a^	3.25 (1.10)^a^	3.13 (1.32)	3.64 (1.16)
Living location	3.68 (1.13)	4.04 (1.21)	3.45 (1.31)	3.72 (1.12)
Life in general	2.58 (1.02)^a^	3.87 (1.11)^a^	3.90 (1.14)	4.07 (1.31)
Average	3.20 (0.70)^b^	3.83 (0.88)^b^	3.99 (0.64)	4.05 (1.04)

Abbreviations: AN, anorexia nervosa; comparison group, adolescents without an eating disorder.

^a^ A significant difference between groups on that specific item, using Mann–Whitney U tests with a Bonferroni‐Holm correction, α < .0125.

^b^ A significant difference between groups, using an univariate ANOVA, α = .05.

### 3.3 Post hoc tests

As the between subjects tests showed differences between groups in satisfaction with the meaningful life domains, we subsequently examined for which specific domains these differences between groups were evident. Because assumptions of normality were violated when looking at individual domains, we decided to conduct six separate Mann–Whitney *U* tests. A Bonferroni–Holm correction was applied to correct for familywise error rate. Therefore, the smallest *p* value was tested against an alpha of.0083; the *p* values following against .01, .0125, .0167, and .025 and the largest against .05. The analyses revealed significantly lower satisfaction with the self (*U* = 747.50, *p* < .001), satisfaction with life in general (*U* = 952.50, *p* < .001) and satisfaction with school experience (*U* = 1822.50, *p* = .012) in individuals with AN compared to individuals without eating disorders. No significant differences between groups were found for satisfaction with living location (*U* = 1856.50, *p* = .019), friendships (*U* = 2189.50, *p* = .387) and family (*U* = 2290.00, *p* = .673).

Finally, to further understand the patterns found in the present study, we post hoc examined whether individuals with AN showing more intense eating disorder symptoms reported less satisfaction with and importance of normative domains compared to individuals with AN showing less intense eating disorder symptoms. Results showed that more intense eating disorder symptoms were indeed related to less satisfaction with normative life domains (EDE‐Q *r* = −.37, *p* = .002); however, such a relationship was absent regarding BMI (BMI *r* = −.20, *p* = .108). No significant relationships were found between symptom severity and the importance of normative life domains (EDE‐Q *r* = .06, *p* = .616; BMI *r* = −.00, *p* = .972).

## 4 DISCUSSION

The current study examined whether adolescents with broadly defined AN differed from healthy weight adolescents without an eating disorder in their satisfaction with and importance of normative life domains. In line with our hypotheses, individuals with AN reported lower satisfaction with normative life domains than individuals without an eating disorder. These results may not necessarily be unexpected, given that eating disorder symptoms were expected to have a negative correlation with satisfaction (Claydon et al., 2020). However, we found no significant difference regarding the importance of these domains between groups. Furthermore, exploratory analyses showed that the overall group difference in the satisfaction measure was primarily driven by individuals with AN reporting lower satisfaction with the self, school experiences and life in general.

These findings are consistent with previous research in a non‐clinical, college student sample (Matthews et al., 2012), in which disordered eating behaviours and weight perceptions were found to be inversely related to satisfaction with normative life domains. To the best of our knowledge, no other study has examined satisfaction with specific normative life domains in relation to eating disorder pathology. Despite the relative dearth of research studying satisfaction with normative domains in individuals with AN, there are a few studies examining satisfaction with life as a whole, generally using the Satisfaction With Life Scale (Pavot & Diener, 1993). Results show that individuals with AN report lower levels of satisfaction with life as a whole than individuals without eating disorders (Garcia et al., 2017; Kitsantas et al., 2003; Magallares et al., 2014). Because individuals with AN included in these studies were all college students or adults with a mean age of 21 or higher, our results extend previous findings by showing that lowered satisfaction with life in general can also be found in adolescents with AN.

In addition, lower satisfaction with the self was found to differentiate between individuals with AN and individuals without eating disorders in the current study (cf. Matthews et al., 2012). However, when asking individuals to rate how satisfied they are with themselves, it is debatable whether the measure taps into satisfaction with a normative life domain or whether it actually assesses self‐esteem. The difference between the groups on this item is in line with the observation that eating disorder pathology is strongly related to low self‐esteem (Brockmeyer et al., 2013; Fairburn et al., 2003). For example, in a longitudinal study, Halvorsen and Heyerdahl (2006) evaluated self‐esteem and satisfaction with life in adults who had been diagnosed with AN during their childhood or adolescence. This study showed that satisfaction with life was reduced in all participants, irrespective of whether participants developed normal eating attitudes or not, and was strongly related to self‐esteem. Because self‐esteem was not independently assessed in the present study, it remains unknown whether the lower satisfaction with normative life domains reported by individuals with AN might be at least partially explained by lowered self‐esteem.

Finally, individuals with AN also reported to be less satisfied with their school experiences than individuals without an eating disorder. This finding is also in line with the study of Matthews et al. (2012), in which satisfaction with school was found to be negatively associated with eating disorder pathology. A possible explanation for this finding is that the strong perfectionism and achievement striving frequently seen in AN (Bardone‐Cone et al., 2007; Fairburn et al., 2003) generates the need to do well in all life areas, including school. However, due to the physical, emotional and social consequences of AN, a lot of individuals with AN are unable to attend school or miss school hours due to treatment appointments (Cook‐Cottone & Lampard, 2017), possibly explaining the lower satisfaction rates.

The present study also showed some unexpected findings. First, it is surprising that individuals with AN did not report lower satisfaction with their friends and family than the comparison group, given earlier findings that individuals with AN report elevated stress related to these domains. For example, previous studies have shown that difficulties with social interactions and friendships may play a role in the development and maintenance of AN (Doris et al., 2014; Treasure & Schmidt, 2013; Westwood et al., 2016). With respect to family functioning, several unhealthy family styles like rigidity, poor communication, low social support and criticism from mothers have also been shown to be related to the development of AN (Ghaderi, 2003; Holtom‐Viesel & Allan, 2014; Krug et al., 2015). Moreover, in two previous studies, both individuals with AN and their parents reported lower satisfaction with their families compared to controls (Fisher & Bushlow, 2015; Laghi et al., 2015). However, two differences between the current study and previous studies might explain the discrepancy between previous and current findings. First, the sample sizes of Fisher and Bushlow (2015) and Laghi et al. (2015) are relatively small (*N* = 44 and *N* = 36, respectively), and in the study of Fisher and Bushlow (2015), participants with other eating disorders than AN were included. Second, in the study of Fisher and Bushlow (2015), individuals with AN were assessed at a mean of 175 days since their first visit to the treatment centre. In the current study, assessment took place at the start of treatment or up to 4 weeks after intake. Thus, it is likely that the participants in the study of Fisher and Bushlow (2015) had a longer duration of illness than the participants in the current study, possibly explaining the differences in satisfaction rates. Unfortunately, no information on the moment of assessment or the duration of illness was provided by Laghi et al. (2015).

Second, despite individuals with AN reporting less satisfaction with normative domains, the between‐subject test of the importance of these domains showed no difference between individuals with AN and those without an eating disorder. The similar importance scores of normative life domains might be explained by the strong perfectionism seen in AN (Bardone‐Cone et al., 2007), generating the need to do well in all life areas. However, the engagement in AN‐related activities and goals potentially reduces the ability to engage in normative domains as much as individuals without AN would, making lower satisfaction scores more likely. Furthermore, even if importance ratings of domains are high, this does not necessarily imply that goals related to that domain are also actively pursued. For instance, a recent study suggests that goals can become ‘frozen’, meaning that goals are not actively pursued and effort is reduced, whereas they remain personally important, and commitment is not relinquished (Davydenko et al., 2019). Thus, for individuals with AN, goals related to normative life domains might have become ‘frozen’, explaining the high importance but low satisfaction rates.

The present study has some important strengths, including the use of a large sample of adolescents with AN and matched comparisons and the use of a domain specific measure to assess satisfaction with and importance of normative life domains. However, six limitations should be taken into account. First of all, the present study used a cross‐sectional design, which does not allow for inferences concerning directionality of the relationships. Future longitudinal studies should examine whether low satisfaction with normative life domains plays a role in the development and/or maintenance of AN, whether satisfaction fluctuates together with the disorder or whether low satisfaction with normative domains is a consequence of AN. Additionally, to study the relevance of the findings for clinical interventions, longitudinal research should examine whether intervening on the relevant individual domains has a positive effect on AN symptoms. Second, an important downside of using a domain specific measure to assess satisfaction with normative life domains is that the use of nomothetic domains may not apply to everyone. Thus, to get a better sense of whether non‐AN‐related domains and goals are important in AN, it would be helpful to examine idiographic measures of meaningful domains and goals, including the number, types and commitment to pursuing these domains and goals. As a third limitation, it should be acknowledged that, although we specifically recruited adolescents without eating problems for the comparison group, this was not verified with a diagnostic interview. However, as post hoc excluding individuals from the comparison group with adjusted BMI lower than 85% (*n* = 2) and/or EDE‐Q scores in the clinically significant range (*n* = 2; cf. Carter, Stewart, & Fairburn, 2001) did not affect the pattern of results, we feel it is safe to draw conclusions about differences between groups. More details on this matter can be found in the supporting information.

Fourth, the inclusion of individuals with atypical AN potentially complicates findings of the current study, in light of possible differences in individuals with typical and atypical AN (Garber et al., 2019; Sawyer et al., 2016). Unfortunately, our current sample size does not allow us to reliably examine differences between individuals with typical and atypical AN. Yet, if the analyses were restricted to the individuals with typical AN, we do find similar patterns in which individuals with AN report lower satisfaction with normative life domains than individuals without an eating disorder. Fifth, the broad age range in the current study (12 to 22 years old) might also complicate our results. However, it is important to mention that 89.2% of all participants were aged 14 to 19 years old and that post hoc excluding participants outside of this age range did not affect the pattern of results. Finally, it is unclear whether there are gender effects in the relation between AN and satisfaction with life domains, as participants in the current study were mostly female (97%). Previous research suggests that disordered eating may be differentially related to life satisfaction across men and women (Zullig et al., 2007). It may therefore be relevant for future research to examine whether satisfaction with normative life domains is also different in men and women with AN.

In conclusion, the present study showed that adolescents with broadly defined AN report lower satisfaction with normative life domains, compared to non‐eating disordered individuals. Specifically, individuals with AN mainly seem to be less satisfied with themselves, with their school experiences and with life in general. These results highlight the value of the unique information found when looking at satisfaction with normative life domains. More longitudinal research is needed, however, to examine whether a decrease in eating disorder symptoms is related to an increase in satisfaction with meaningful domains. Finally, it would be important to examine the proposed causal influence of low satisfaction with normative life domains on the persistence of eating disorder symptoms in individuals with AN. Therefore, a critical next step would be to experimentally increase satisfactory engagement with goals related to normative life domains and to see whether this would also result in a decline of symptoms. If so, this would not only attest to the relevance of low satisfaction with meaningful life domains in the persistence of AN but would also point to normative life domains as a relevant target for treatment.

## CONFLICT OF INTEREST

The authors have no conflict to declare.

## Supporting information


**Data S1.** Supporting information

**Figure S1.** Supporting information

**Figure S2.** Supporting information

**Figure S3.** Supporting information

**Figure S4.** Supporting information

**Figure S5.** Supporting information

**Figure S6.** Supporting information

**Figure S7.** Supporting information

## Data Availability

The datasets generated during and/or analysed during the current study are available from the corresponding author on reasonable request.
