# Supplementary material for: Low satisfaction with normative life domains in adolescents with anorexia nervosa
Source: Clin Psychol Psychother. 2021 Mar 2;28(5):1266–74. doi: 10.1002/cpp.2574 (PMC8596741; doi:10.1002/cpp.2574)

**Descriptive statistics: EDE-Q**

|  | | | | | |
| --- | --- | --- | --- | --- | --- |
|  | | **EDE-Q** | | | |
|  | | **Comparisons** | | **AN** | |
| Valid |  | 69 |  | 69 |  |
| Missing |  | 0 |  | 0 |  |
| Mean |  | 1.296 |  | 4.161 |  |
| Std. Error of Mean |  | 0.133 |  | 0.134 |  |
| Std. Deviation |  | 1.104 |  | 1.115 |  |
| Range |  | 4.909 |  | 4.591 |  |
| Minimum |  | 0.000 |  | 1.227 |  |
| Maximum |  | 4.909 |  | 5.818 |  |
|  | | | | | |

**Distribution and Q-Q Plots: EDE-Q**

**Comparison Group**


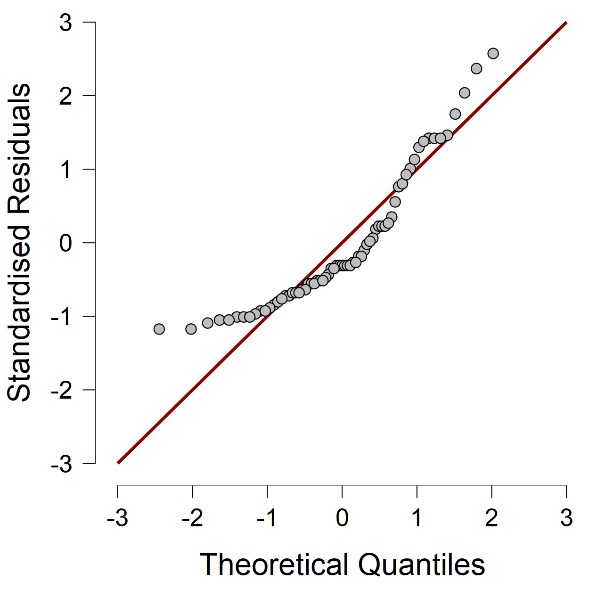


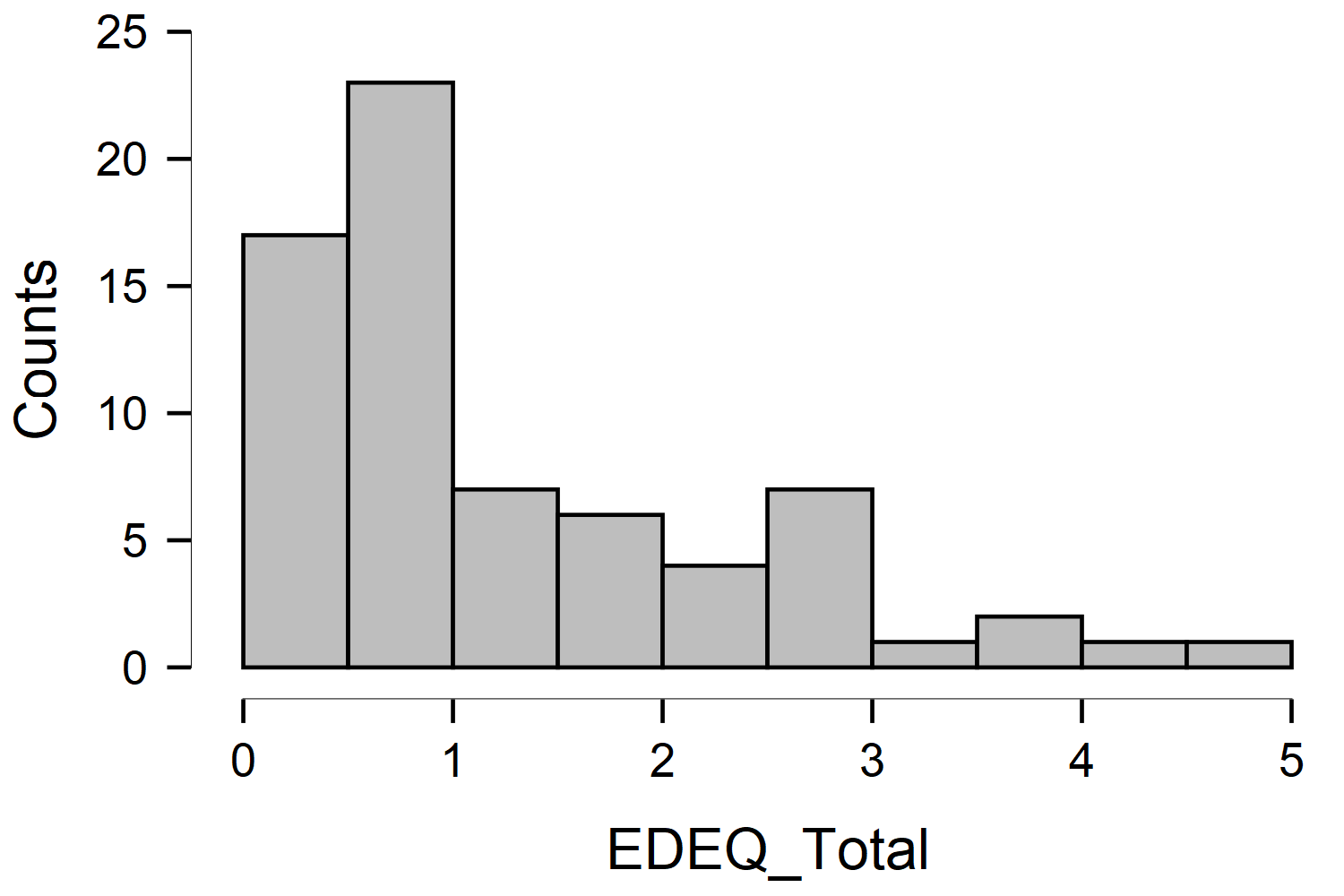


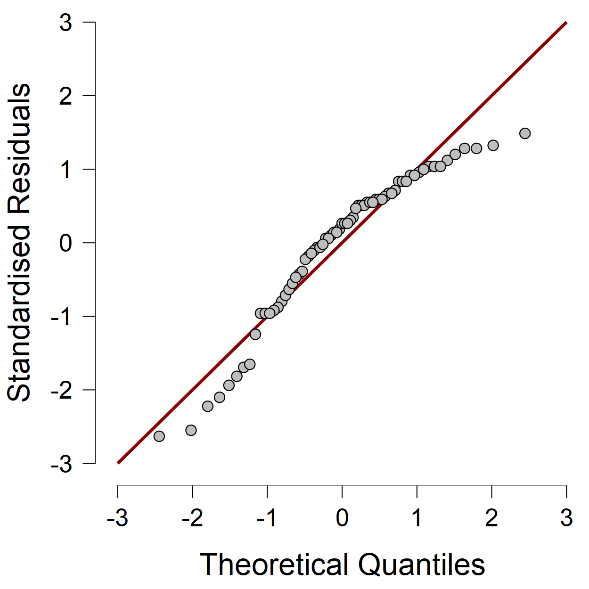

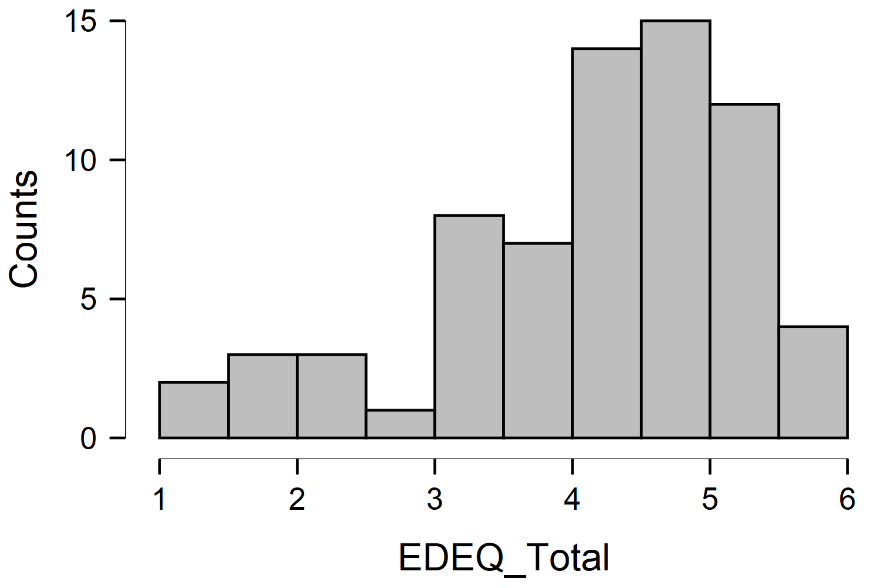
**Individuals with AN**

**Descriptive statistics: Adjusted BMI**

|  | | | | | |
| --- | --- | --- | --- | --- | --- |
|  | | **Adjusted BMI** | | | |
|  | | **Comparisons** | | **AN** | |
| Valid |  | 69 |  | 69 |  |
| Missing |  | 0 |  | 0 |  |
| Mean |  | 102.868 |  | 84.691 |  |
| Std. Error of Mean |  | 1.158 |  | 1.464 |  |
| Std. Deviation |  | 9.618 |  | 12.160 |  |
| Range |  | 45.267 |  | 64.757 |  |
| Minimum |  | 84.167 |  | 61.657 |  |
| Maximum |  | 129.434 |  | 126.414 |  |
|  | | | | | |

**Distribution and Q-Q Plots: Adjusted BMI**


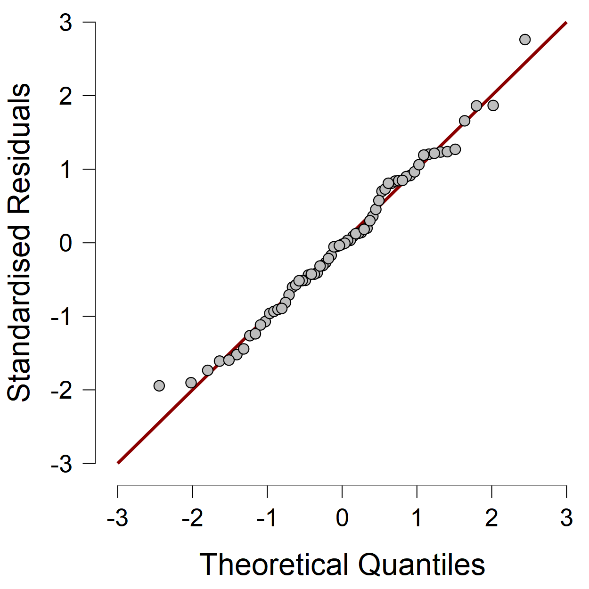
**Comparison Group**


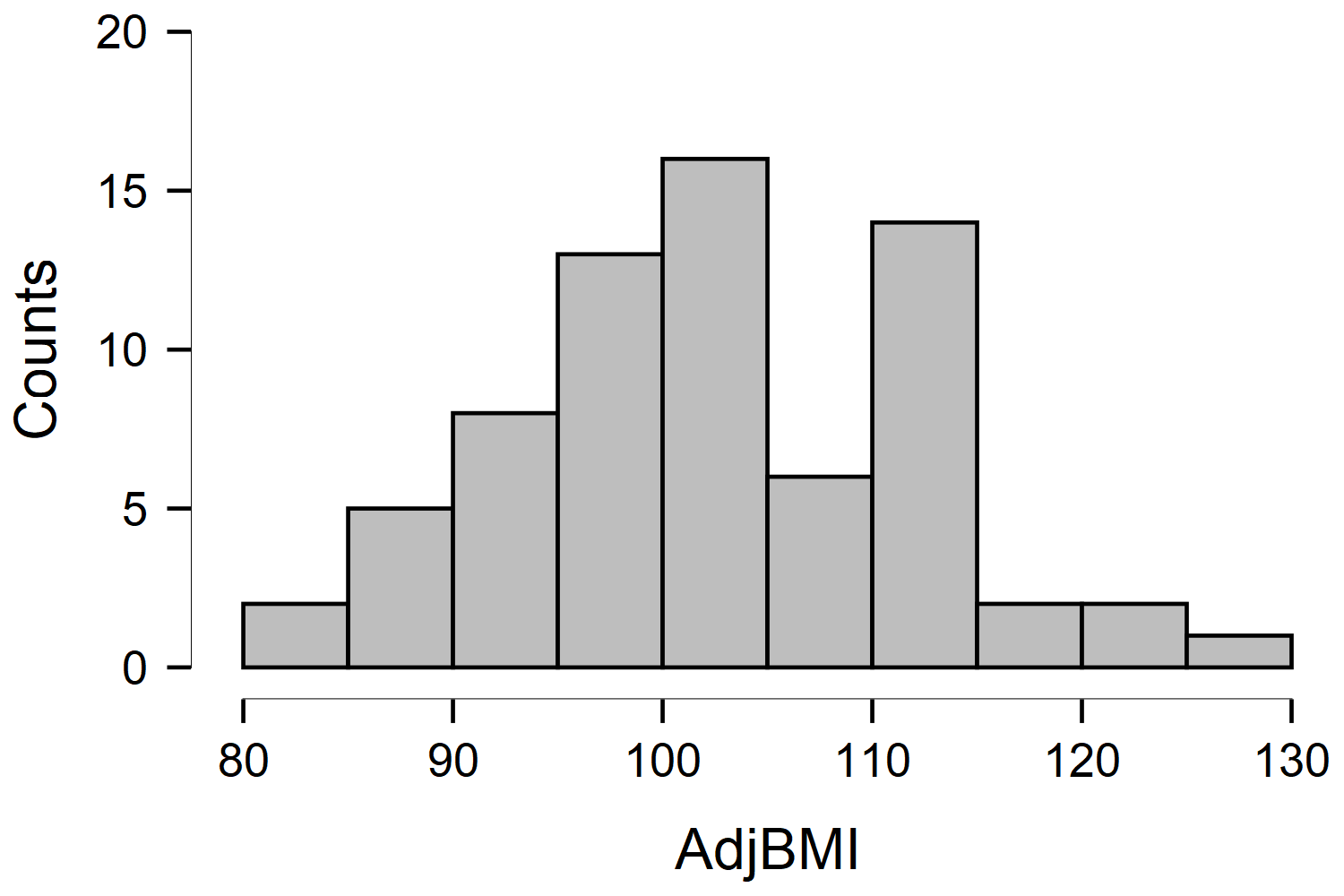


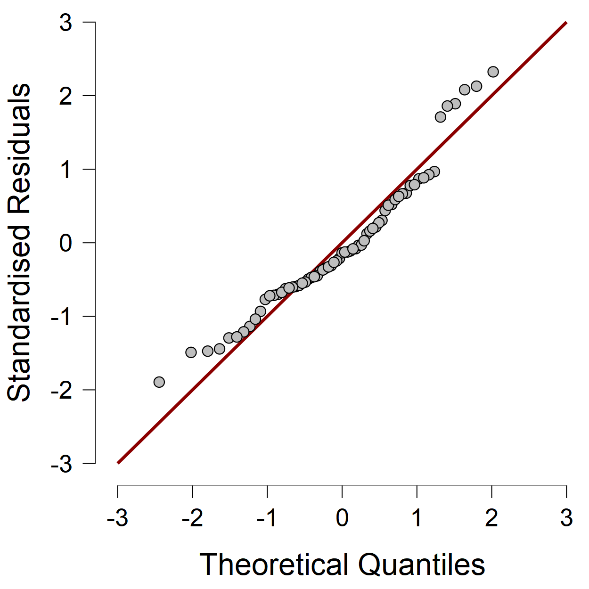
**Individuals with AN**


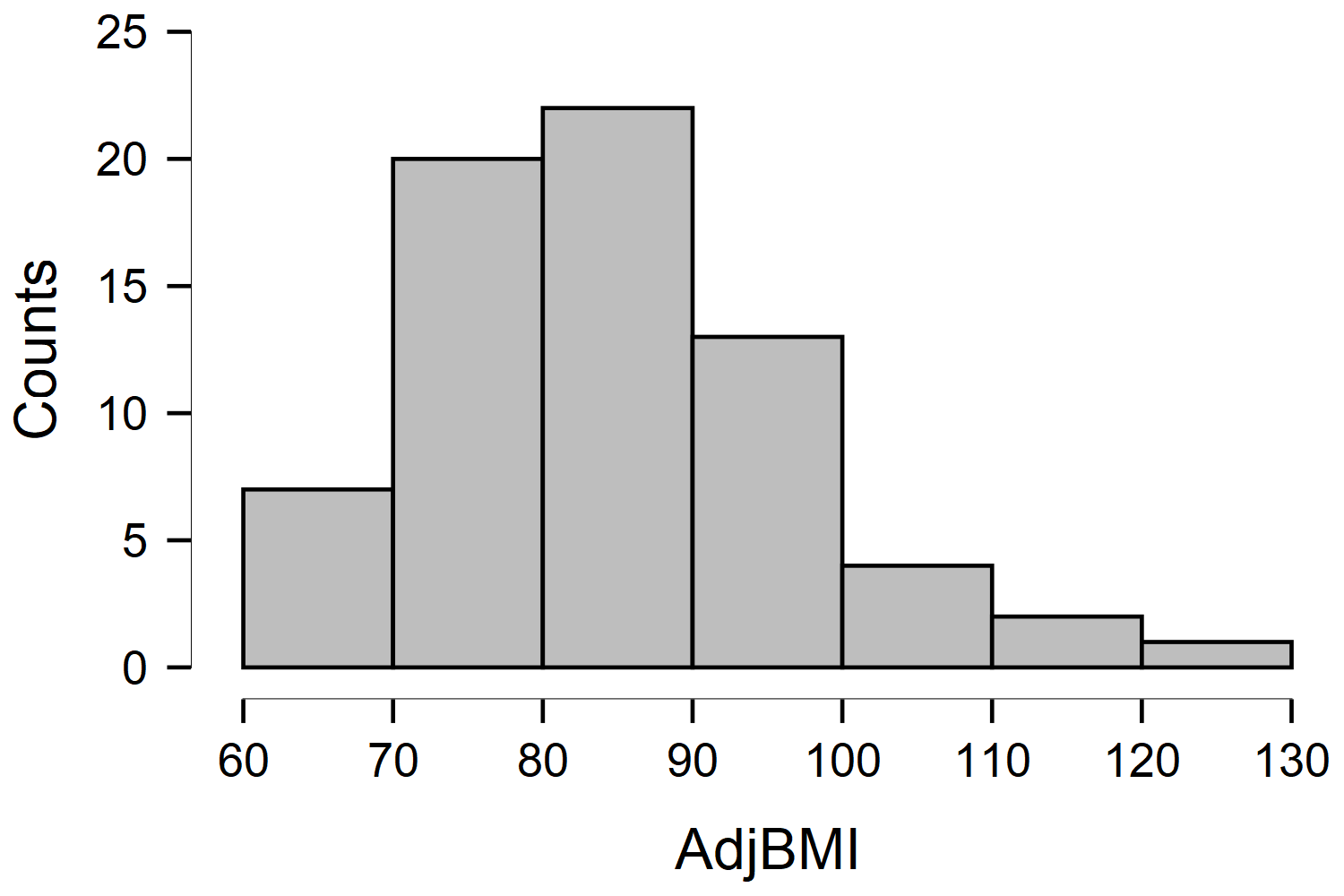


**EDE-Q – Adjusted BMI**


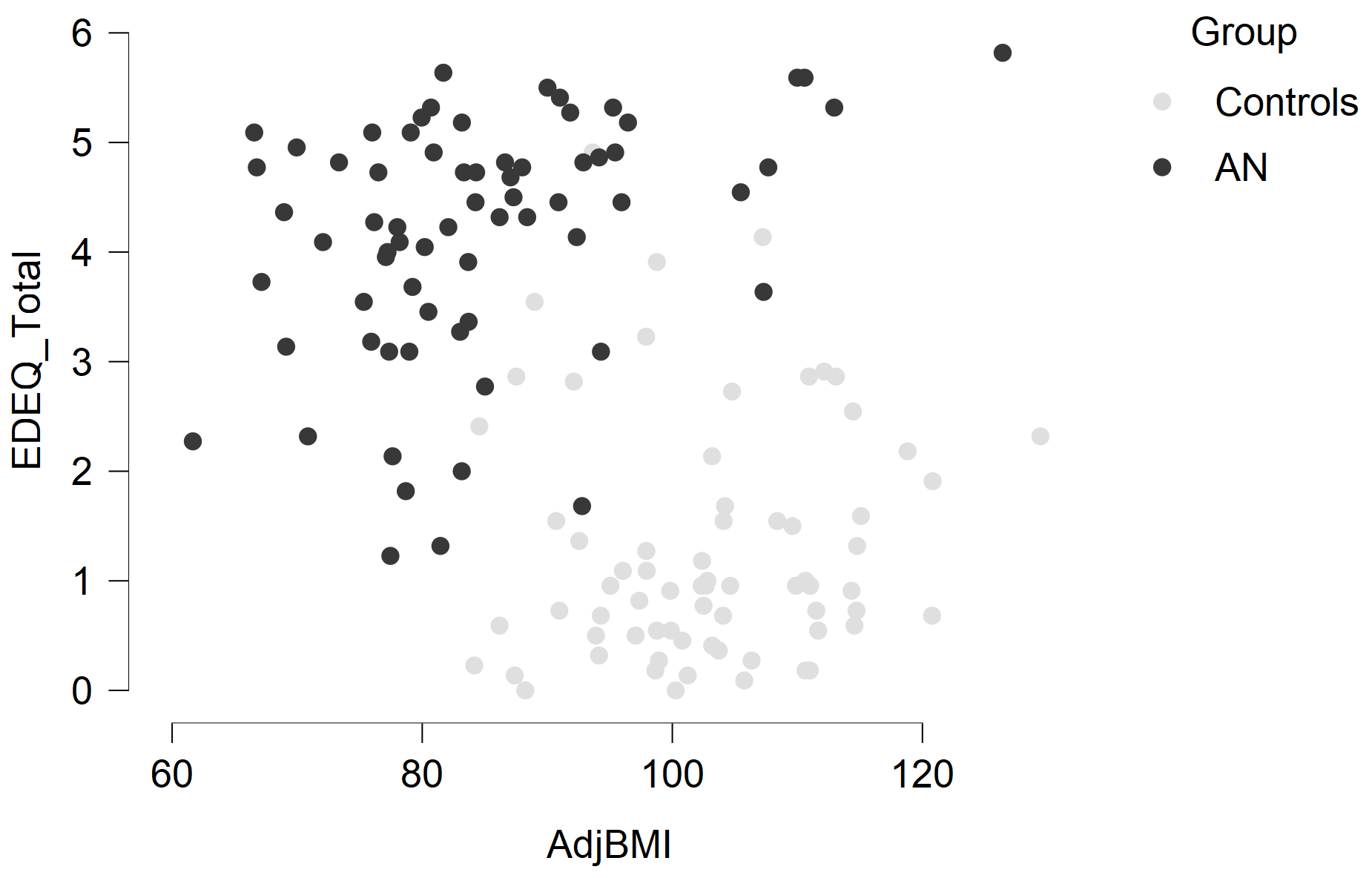

Supplement: Supplementary file 1 — Data S1. Supporting information [file CPP-28-1266-s004.docx]
