# Supplementary figures and images for: Low satisfaction with normative life domains in adolescents with anorexia nervosa
Source: Clin Psychol Psychother. 2021 Mar 2;28(5):1266–74. doi: 10.1002/cpp.2574 (PMC8596741; doi:10.1002/cpp.2574)

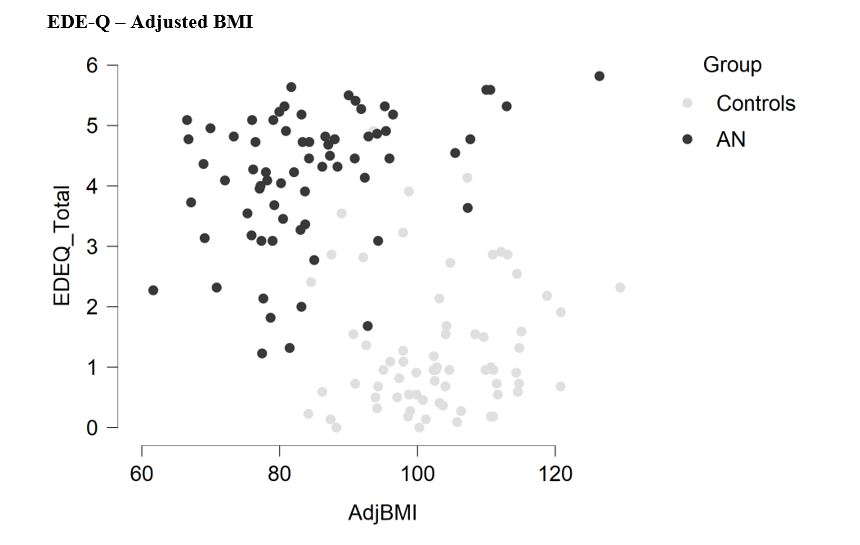

Supplement: Supplementary file 2 — Figure S1. Supporting information [file CPP-28-1266-s003.JPG]

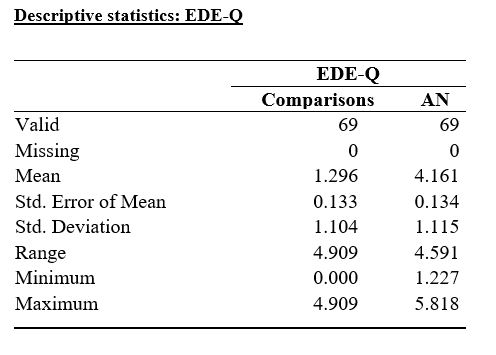

Supplement: Supplementary file 3 — Figure S2. Supporting information [file CPP-28-1266-s002.JPG]

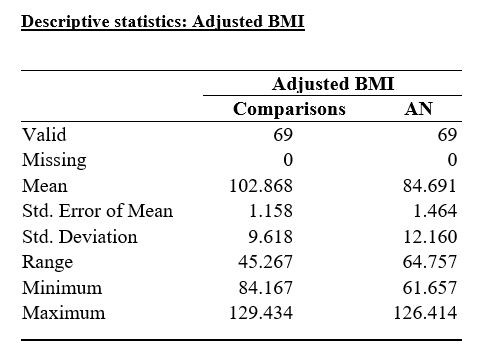

Supplement: Supplementary file 4 — Figure S3. Supporting information [file CPP-28-1266-s006.JPG]

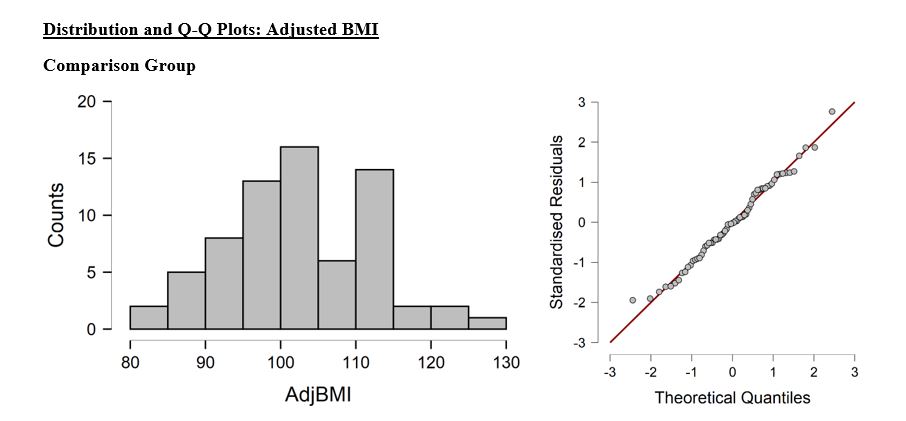

Supplement: Supplementary file 5 — Figure S4. Supporting information [file CPP-28-1266-s005.JPG]

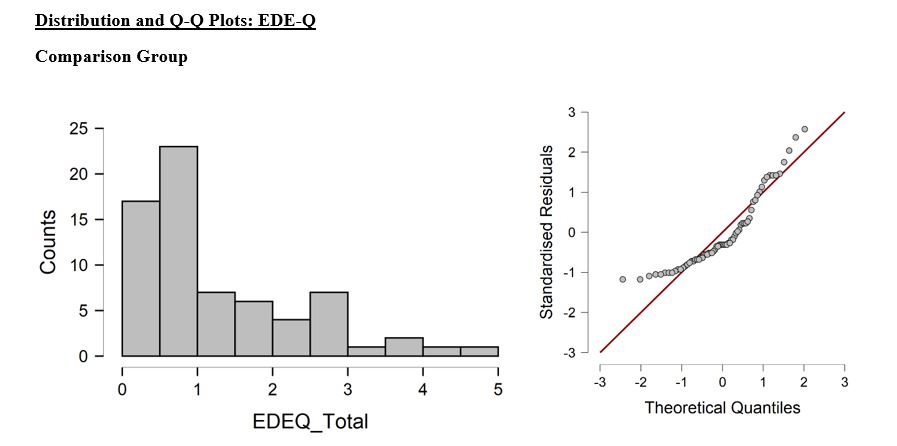

Supplement: Supplementary file 6 — Figure S5. Supporting information [file CPP-28-1266-s007.JPG]

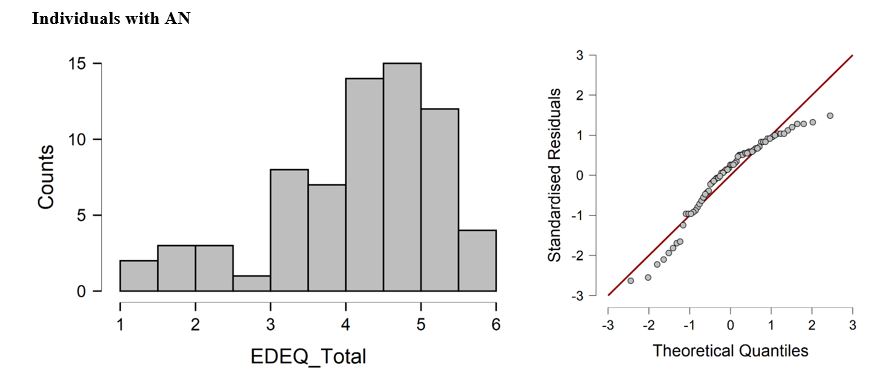

Supplement: Supplementary file 7 — Figure S6. Supporting information [file CPP-28-1266-s001.JPG]

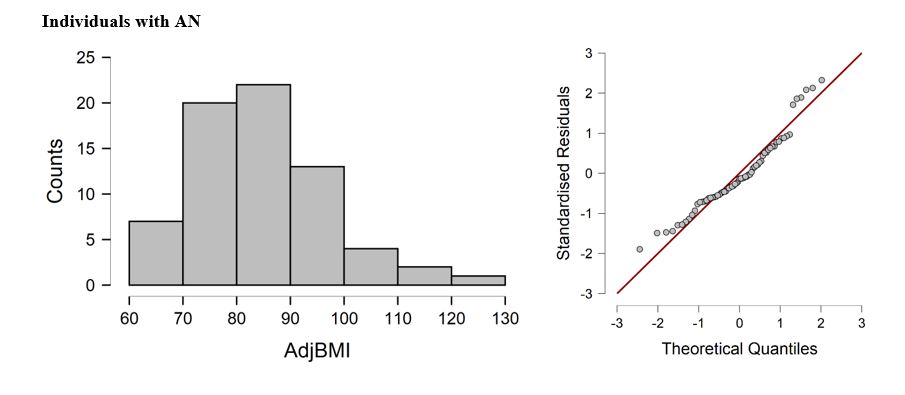

Supplement: Supplementary file 8 — Figure S7. Supporting information [file CPP-28-1266-s008.JPG]
